# A biosafety level 2 virology lab for biotechnology undergraduates

**DOI:** 10.1002/bmb.21080

**Published:** 2017-07-30

**Authors:** Sigal Matza‐Porges, Dafna Nathan

**Affiliations:** ^1^ Department of Biotechnology Hadassah Academic College Jerusalem Israel

**Keywords:** Virology, PCR, ELISA, MulV

## Abstract

Medical, industrial, and basic research relies heavily on the use of viruses and vectors. Therefore, it is important that bioscience undergraduates learn the practicalities of handling viruses. Teaching practical virology in a student laboratory setup presents safety challenges, however. The aim of this article is to describe the design and implementation of a virology laboratory, with emphasis on student safety, for biotechnology undergraduates. Cell culture techniques, animal virus infection, quantification, and identification are taught at a biosafety level 2 for a diverse group of undergraduates ranging from 20 to 50 students per group. © 2017 by The International Union of Biochemistry and Molecular Biology, 45(6):537–543, 2017.

## Introduction

Viral infection and viral epidemics attract considerable public attention 1 and the use of viral vectors in clinical and basic research is growing every year 2. Consequently, it is important to teach the practical aspects of viral manipulation to life science undergraduates.

Students need to learn safe handling of viruses, viral production, maintenance of infectivity, and storage of viruses. Upon graduation, students with basic working knowledge in virology may have an edge over their peers who are not familiar with such methods. From an educational perspective, combining hands‐on experience with an in‐class virology course allows the instructor to expand certain topics that are covered in class and connect them to research in biomedical areas 3, 4.

Laboratory work in virology requires students to operate under biosafety level 2 (BSL2) conditions (see information box). Because of the technical and safety issues, teaching virology in a practical laboratory setup poses various challenges. Nevertheless, undergraduates in biotechnology and medical science laboratories at Hadassah Academic College in Jerusalem take this lab as part of the mandatory curriculum. Roughly 20–50 students participate in the laboratory class simultaneously. Students work in pairs under personal supervision and use biohazard materials in biological hoods without endangering themselves and their colleagues. In the lab, students encounter a variety of relevant methods including ELISA, diagnostic PCR, quantification using reporter gene, and more. This article details the laboratory design, management, and student evaluations.

Biosafety Level 2 (BLS2): All work involving infection with MulV requires students to comply with following guidelines: listen to a safety lecture; wear protective clothes, gloves, and glasses; pour liquid waste into bleach containers; autoclave any liquid and solid waste; use virus‐containing tubes only within biological hoods; and centrifuge the virus in special screw‐top sealed tubes.

## Materials and Methods

### Cell Lines

Murine fibroblast NIH3T3 and Monkey kidney Vero cells were cultured in Dulbecco's Modified Eagle's Medium (DMEM, Sigma Aldrich, St Louis, MO, USA) supplemented with 10% fetal calf serum (FCS, Sigma Aldrich), penicillin (100 U/ml), and streptomycin sulfate (100 μg/ml) at 37°C in 5% CO_2_. Vero cells were kindly provided by Dr A. Panet (Department of Virology, The Hebrew University, Hadassah School of Medicine, Jerusalem, Israel).

### Viruses and Bacteria

*Escherichia coli* lysogenic strain bearing bacteriophages λ *c*I857, carrying a temperature‐sensitive repressor mutation 6 and *Escherichia coli* host strain ER1647 (Novagen corp) were grown in Liquid Broth (LB) medium contained 1% Trypton (Bactlab ‐ Becton Dickinson), 0.5% NaCl, and 0.5% yeast extract (Bactlab ‐ Becton Dickinson).

Ecotropic defective MulV vector expressing the *Lac*Z gene (β‐Galactozidase) was produced from NIH3T3 Packaging cells 5. The virus includes a sequence encoding a modified 13‐galactosidase and the simian virus 40 nuclear location signal, nls‐LacZ 7.

HSV 17+/pR20.5/5 contains the β‐galactosidase (β‐Gal) gene, which is under the control of the Rous sarcoma virus (RSV) promoter, and the green fluorescent protein (GFP) gene, which is under the control of the cytomegalovirus (hCMV) promoter. The virus was kindly provided by Dr A. Panet (Department of Virology, The Hebrew University, Hadassah School of Medicine, Jerusalem, Israel). To propagate the virus, Vero cells were infected and the virus was harvested from the cell pellet, purified on a 10% sucrose cushion (Sigma Aldrich), and titrated by plaque assay on Vero cells 8.

### Laboratory Procedures

#### Viral Quantification by Plaque Assay

*Escherichia coli* lysogenic for λc1857 were grown with gentle shaking in Liquid Broth (LB) medium at 30°C for overnight. Thirty microliters of the *E. coli* culture was inoculated into 3 ml LB at a starting optical density of 0.05 measured at 590 nm (OD_590_) and grown at the same conditions to an optical density of 0.2. The culture is then moved to 42°C. OD and a 100 μl sample is taken every 20 min for 75 min. Each 100 μl sample is decimal diluted from 10^−1^ to 10^−7^ and 3 prechosen dilutions from each time point were prepared for plaque assay by mixing with 20 μl 0.1 M ${\text{MgSO}}_{4}^{-}$ and with 100 μl of *E. coli* strain ER1647grown overnight in Liquid Broth (LB) medium at 37°C. Samples were incubated at room temperature for 10 min and mixed with LB soft agar (0.6%, Novamed corp.) at 56°C, and plated on LB plates (Novamed corp), for overnight incubation at 37°C.

#### MulV Quantification

NIH3T3 cells (500,000) were seeded in 5 cm^2^ plates in 1 ml DMEM, for 24 hr and then infected with 10^−3^–10^−7^ dilutions of MulV in 1 ml DMEM, with 4 μg/ml Polybrene (sigma Aldrich) and no fetal calf serum (FCS). Two hours postinfection, 10% FCS and DMEM were added to achieve a total volume of 5 ml, and cells were incubated for a further 48 hr at 37°C in 5% CO_2_. After 48 hr, NIH3T3 cells were fixed using 0.2% gluteraldehyde (Sigma Aldrich), 2% formaldehyde (Sigma Aldrich), 2 mM MgCl_2_ in 1× PBS, for 10 min at room temperature and stained with 0.1% 5‐bromo‐4‐chloro‐3‐indolyl‐β‐d‐galactoside (X‐Gal, Promega). The number of blue cells was counted in five 1 mm^2^ randomly selected squares and the titer of the virus was calculated based on the plate area and the dilution of the virus.

#### Immunocytology Assay

Vero cells were seeded in 96‐well plates (1 × 10^4^ cells per well) in DMEM, for 24 hr and then infected with 10^−3^–10^−7^ dilutions of HSV‐(RSVβ‐gal) in 50 μλ DMEM, with no FCS. One hour postinfection, 10% FCS and DMEM were added to achieve a total volume of 100 μl, and cells were incubated for a further 48 hr at 37°C in 5% CO_2_. After 48 hr, Vero cells were fixed using 100 μl 3.8% formaldehyde in PBS, for 10 min at room temperature, washed three times with 1× PBS and blocked for 30 min at room temperature with 100 μl 1% BSA in PBS (blocking solution). Cells were washed with 1× PBS 0.02% Tween 20 and treated with 50 μl polyclonal Rabbit anti HSV‐1 glycoproteins (Biotest) diluted 1:100 with blocking solution for 1 hr at room temperature. After three washes with 1× PBS 0.05% Tween 20, cells were treated for 30 min with 50 μl goat anti rabbit IgG HRP conjugated (Biotest), and washed again. One hundred microliters of TMB (3,3′,5,5′‐tetramethylbenzidine; MW = 240.4) (Biotest) substrate was added and the absorbance of the soluble blue product was measured at 650 nm. As a positive control Vero cells were infected with 10^−3^ dilution of HSV‐(RSVβ‐gal) with 10 μM of acyclovir (Sigma Aldrich), a known anti‐HSV agent.

#### Human Cytomegalovirus (hCMV) Strain Analysis

hCMV DNA was purified from HFF cell supernatant by QIAamp DNA Blood mini Kit (QIAGEN), and the gB sequence was amplified by PCR (HotStarTaq Polymerase Kit, QIAGEN) using two oligonucleotide primers: 5′‐TGG AAC TGG AAC GTT TGG C‐3′; 5′‐ GAA ACG CGC GGC AAT CGG‐3′. The PCR product was cleaned by an analytical column (QIAquick PCR Purification Kit, QIAGEN), and cut with the RsaI and HinfI restriction enzymes (Promega Corp.), to determine the virus strain 9.

### Academic Prerequisites and Relevant Background

The lab requires prior knowledge in virology and general familiarity with laboratory and cell culture techniques. Students learn these skills by taking prior courses and labs in their first and second year of the program.

The course in virology is taught in parallel, or prior, to the virology laboratory and is a prerequisite for the lab. Class materials include an introduction to animal‐virus classification, structure, and life cycle and methods for the preparation of virus stocks, virus concentration, quantification, and characterization (Supporting Information).

### Laboratory Design, Choice of Viruses, and Safe Lab Procedures

The lab consists of two parts. The first part focuses on virus infection and quantification methods. The second part concentrates on how to identify viruses using molecular methods. The goal of the first part is to teach students ways of counting viruses, and compare direct methods (in which the number of infective units is counted) to methods that count the total number of viral particles (virions). Students learn how to make serial dilutions of the virus, and infect cells with different dilutions to count viruses efficiently. They also learn how to calculate the multiplicity of infection (MOI) from viral stocks with known titers.

While choosing the appropriate virus with which to work, it is important to bear in mind that undergraduates are not experienced in laboratory work. Hence, the risk of work related accidents is high 10, 11. In light of this concern, is it important to select a virus that poses the lowest biosafety risk, within the BSL2 guidelines. Another important safety aspect of the lab is a low student‐to‐instructor ratio. The instructors are all graduate students, experienced with cell culture techniques and handling of viruses.

Most of the work is carried out with a replication defective, nonreplicative, ecotropic‐MulV‐based viral vector expressing β‐galactosidase 7, 12. This vector does not contain any viral structural protein genes. It infects only mouse cells and poses a low safety risk.

Students also use a recombinant replicative HSV‐1 virus (Herpes Simplex Virus type 1) 8. This vector poses a higher safety risk. Hence, students work with it only after gaining experience with the MulV vector under BSL2 conditions and personal supervision.

## Laboratory Content

The overall design of the lab is shown in Table 1. To teach safety procedures gradually, the first lab focuses on counting viruses using the Plaque Assay method. Students titrate bacteriophage Lambda after the induction of a lysogenic *E. coli* harboring a temperature‐sensitive (ts) mutant, Lambda prophage. This experiment is performed in a traditional lab space under BSL1 conditions. This stage introduces working techniques with viruses while teaching students the principles of plaque counting. The bacteriophage harbors a ts mutation in the gene coding for the Cl repressor (λc1857). The phage is lysogenic at 30°C, but turns lytic at 42°C due to inactivation of the ts repressor. Following induction, the bacteriophage replicates, causes lysis, and releases to the medium. Students grow the bacteria to logarithmic stage, switch on the lytic cycle by moving the bacteria to 42°C, and follow bacterial lysis indirectly by recording the optical density of the medium. To determine the number of released phage particles, different dilutions are prepared from the medium and are used to infect an indicator bacteria (*E. coli* ER 1647) that is plated in soft agar. The number of plaques is counted after 24 hr incubation at 37°C. The kinetics of phage production is compared to the lysis kinetics.

**Table 1:** Virology lab plan and procedures

Lab number	Lab topic	Techniques involved
1	Bacteriophage life cycle	Growing lysogenic bacteria, moving bacteriophage into a lytic cycle, and monitoring growth using optical density measurements and focal point counting.
2	Animal viruses, infection with a virus containing a reporter gene. Using reporter gene to determine titer.	Infection test for defective MulV: identifying infected cells using β‐galactosidase activity assay.PCR reaction for CMV identification.Counting bacteriophage lysis focal points.
3 (48 hr after lab 2 + submit lab report 1)	Animal viruses. Infecting Vero cells with HSV‐1.	HSV‐1 cell infection.Identification and counting of cells infected with MulV.
4 (48 hr after lab 3)	Immunocytology. ELISA for cell culture infected with HSV‐1. Methods for viral identification ‐ I	Immunocytology‐cell culture ELISA to test for HSV‐1 infection.Cleaning PCR product from lab number 2 using agarose gel.
5	Methods for viral identification – II	Restriction analysis of PCR product, viral species identification based on PAGE results.Lab summary

The second and third labs focus on working with animal viruses, including cell infection and viral quantification in cell cultures. First, the students, working in pairs, learn to observe animal cells in culture by inverted microscope under BSL2 conditions in biological hoods. Each pair works in the hood in a successive fashion under the careful supervision of the instructor. Students learn different methods for counting the number of infective units per milliliter for different types of viruses and differentiate between infective and noninfective viruses before choosing the appropriate counting method. Students receive NIH3T3 cells that were plated a day earlier and perform the infection themselves. Every pair of students works with an instructor for 15–20 min in the biological hood. Students infect the cells with an MulV vector expressing the *E. coli β‐gal* gene. Forty‐eight hours postinfection (lab3, Table 1), students fix the cells and stain them by adding X‐gal. Students count the number of blue cells per square millimeter using light microscopes and calculate the number of infective units on the plate.

The second lab poses an organizational challenge: Each pair of students works in a sterile biological hood for 15–20 min, under personal supervision. Each class includes 17–25 pairs of students. To maximize efficiency, the lab is divided into four posts—three work stations and a lecture area. The lecture, given twice during the second lab period, provides theoretical background on methods for counting cells, transfection, and infection approaches, and practical guidelines for selecting the right method or approach under different circumstances. In addition, the lecture covers technical aspects of cell infection by animal viruses (e.g., cell confluence, replicative cell status and optimal adsorption conditions). In the other two stations, students either count the number of foci formed in lab1 (Table 1) or start a virus identification procedure with a PCR of a DNA extracted from cells infected with the human cytomegalovirus (hCMV).

One of the goals of the lab is to teach students molecular diagnostics methods (serological methods for viral analysis are taught in a preceding immunology lab). Students use PCR to identify an hCMV strain. The primers are homologous to the gB glycoprotein sequence of the virus 9. In the fourth lab, students use an analytical column to clean their PCR product. The eluted PCR product is visualized by agarose gel electrophoresis. In addition, theoretical background regarding methods for the detection of viruses in samples is given during this lab and is combined with information on the importance of hCMV detection in the serum and amniotic fluids of pregnant women.

**Figure 1:**
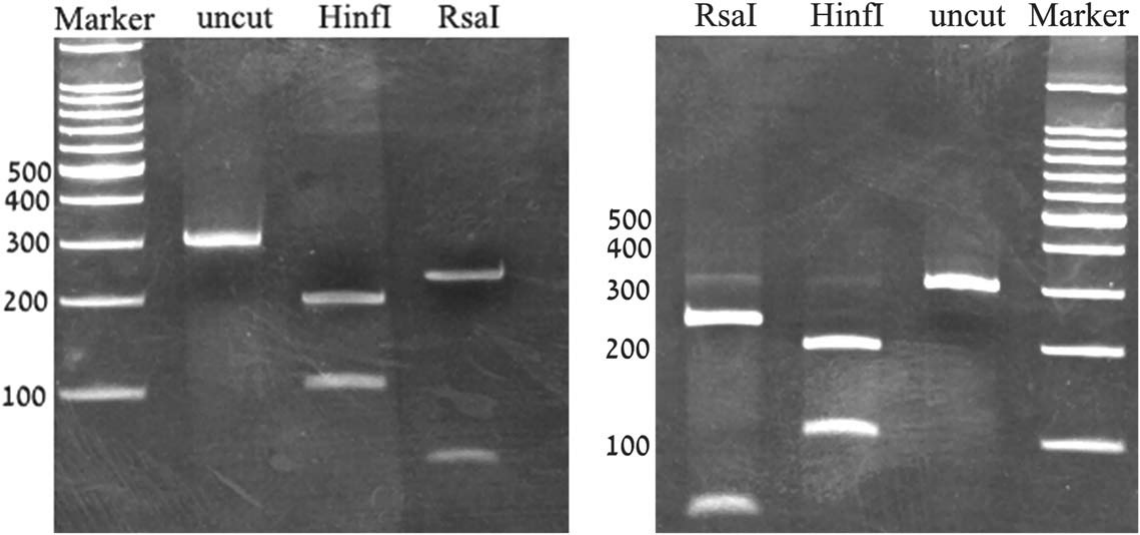
Representative students results of CMV restriction analysis. 8% polyacrylamide gel electrophoresis results of uncut, RsaI, and HinfI restricted PCR product of the cytomegalovirus gB DNA. Two representative student results are shown.

In the fifth lab restriction enzyme analysis of the hCMV, DNA is conducted based on a paper by Chou and Dennison 9. DNA is cut with the restriction enzymes RsaI and HinfI and the fragments are separated by an 8% polyacrylamide gel electrophoresis that allows the separation and detection of fragments ranging from 60 to 200 bp. hCMV strains can be differentiated based on the pattern of restriction fragments formed by RsaI and HinfI. The importance of detecting different viral strains in research and clinic is discussed. Students compare their findings to those reported by Chou and Dennison and use the results to determine the strain they used in the lab. In this way, students also learn about the importance of comparing their results to scientifically published data. Representative results are shown in Fig. 1.

The last topic covered during the third and fourth labs is quantification of the HSV‐1 virus using an immunocytological method and the enzyme‐linked immunosorbent assay (ELISA) detection technique 13. As part of their second‐year requirements, students take a course and lab in immunology, where they learn about ELISA in theory and practice.

**Figure 2:**
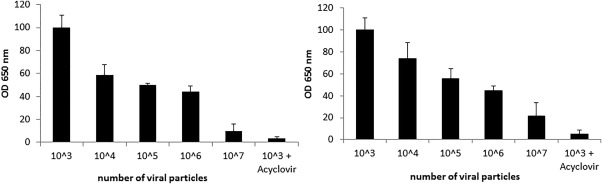
Representative student results of HSV‐1 quantification using ELISA. Readouts of Vero cells infected with different concentrations of HSV‐1 virus. Two representative student results are shown. Students repeat each data point in triplicates and report the average value ± standard deviation from the mean.

Students receive Vero cells that were plated in 96 well plates a day earlier. They infect the cells with different titrations of HSV‐1 virus in a biological hood under the same conditions described in the second lab settings (lab3, Table 1). Forty‐eight hours postinfection, the lab technicians fix the cells with formaldehyde (in a chemical hood) and wash the cells to prepare them for immunocytology. Working on their bench tops, students add the blocking solution, primary and secondary antibodies, and a substrate, and then read their results in an ELISA reader (lab4, Table 1). This procedure teaches students how to calculate the virus concentration in the stock solution and use it for calculation of the MOI in following experiments. Representative results are shown in Fig. 2.

In the last lab, students receive a calculation assignment related to viral infection in culture (supplementary material). This assignment requires students to review their work and to manifest their ability to design the quantitative aspects of an experiment. The assignment serves as part of the student's final grade.

### Evaluating Student Understanding

The final grade is based on four criteria. First, before each lab students take a short quiz that tests their preparation and their initial understanding of the background material, they had to read.

A second evaluation parameter is the students' laboratory reports. Three lab reports are required by the end of the course. The first, detailing the number of plaques relative to the decrease in the optical density of the bacteria. The second, describing the infection of NIH3T3 cells with MulV and the detection of HSV‐1 infected Vero cells by ELISA. The third lab report documents the detection of hCMV by molecular methods. The average grade of these three reports accounts for 25% of the final lab grade.

Students are also evaluated for their ability to calculate and plan different aspects of the experiments by solving an in‐class quantitative assignment. This assignment is given at the end of each lab and is also worth a quarter of the final grade.

Each student also receives an independent evaluation from the lab instructors. Each of the four evaluation aspects accounts for 25% of the students' final grade.

### Student Evaluation of the Lab

Hadassah Academic College holds anonymous student evaluations for every course. Evaluation forms consist of 8 criteria to be scored from 1 (not satisfied) to 5 (very satisfied). Students assess the course instructor, the course design and structure, and the educational contribution of the course in their opinion.

Of the 8 criteria, we chose 3 categories that are specifically relevant to the evaluation of the virology lab: contribution of the lab to the knowledge of the topic, the variety of lab techniques that were taught and course satisfaction.

**Figure 3:**
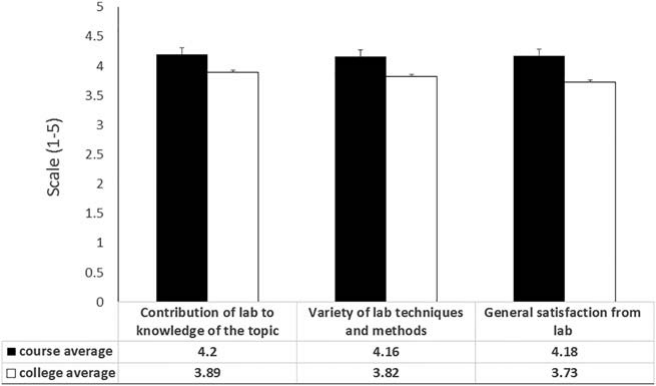
Student evaluation of virology lab compared to college average. The average lab score for each statement (BLACK) is compared to the average college score for the same statement (WHITE). Standard error (SE) is calculated for all mean values. *p* < 0.05 using excel T.TEST function.

Two hundred and thirty students from six different virology labs evaluated the course in the years 2015–2016. A representative evaluation is presented in Fig. 3. The average score for each criterion (BLACK) is compared to the average college score for the same criterion (WHITE). As can be seen, the lab received higher scores than the college average on each of the three criteria and students expressed greater overall satisfaction from the laboratory than the college average for other labs at Hadassah Academic College.

Part of the college questionnaire allows for students to add written remarks on the “highlights” and “lowlights” of the course they are evaluating. Examples of positive remarks included “practical course that contributed a lot to understanding the theoretical one,” “I learned a lot from the lab, gave me a lot of knowledge,” “very enriching,” “the course helped me understand the material.” Critical commentary centered primarily on the fact that the amount of work required of the students was disproportionate to the number of course credits, as can be seen in these examples: “The course requires a lot and the number of credit units is not enough compared to the time and effort invested in it,” “The course requirements are 3 times higher than the number of credit units.” The number of laboratory credit units is determined by the Council for Higher Education and cannot be changed by the college despite student frustration.

## Discussion

Many studies emphasize the importance of combining laboratory courses with theoretical courses to attain deeper student understanding and higher student satisfaction 14, 15. Combining a laboratory course in virology with the in‐class course is important not only for this reason but also because students should be familiar with BSL2 working conditions before they participate in any work involving animal viruses. Because of the relatively higher risk of working with animal viruses, it is important that students learn the subject formally in their undergraduate studies, rather than on the job when they encounter the need to work with viruses.

The main obstacles to teaching a laboratory course on animal viruses are the requirements for highly trained instructors and adequate facilities, including biological hoods in aseptic rooms. Hadassah Academic College relies on graduate students from nearby universities and medical centers as teaching assistants for the course. This interaction has been productive and useful for many years, allowing college undergraduates to meet graduate students from advanced research programs during their studies. The professional laboratory staff is also well trained in tissue culture techniques and provides the general year‐round support for maintenance of cell lines and tissue culture rooms.

Student safety and personal mentoring are fundamental to any teaching laboratory for science undergraduates. The work in biological (aseptic) hoods is central to any tissue culture work and is continuously used in 2 out of the 5 laboratory meetings in the course. The lab work station design allows for personal supervision of students work by a biological hood even though there are only 4 hoods and an average of 35 students per class. By designing 2 or 3 experiments that intertwine with each other during different periods of the lab, a potential bottle‐neck problem is avoided, while still allowing students to learn invaluable techniques by performing the experiments themselves in an individual, supervised way. The rotation between different work stations allows for experiments that continue from one lab to the next, providing students with a sense of an ongoing experiment as will be the case in future work. This design necessitates the use of a variety of experimental methods. It requires students to be well prepared for a few experiments in one lab section, and to keep track of their work from one period to the next. Therefore, this lab course represents an advanced stage in a student's learning program and can also be given as an advanced‐program course.

The virology lab course detailed in this article is organized such that students start their work using practices they have already encountered in other instructional labs and move on to use methods that are specific to research in virology. Although there are only 5 lab periods in this course, the transition between familiar organisms/practices to new ones is necessary for a few reasons. First, it allows students to begin the lab with a familiar topic and known materials and microorganisms. This familiarity may ease off the tension arising from working with viruses that some students may experience. Second, it allows the instructors to identify any potential risk factors related to work habits or student interaction in the lab before working at a higher safety level. The variety of cell lines, viruses, and experimental methods in this lab show students that different types of experiments are not limited to a specific course subject, such as molecular or cell biology. Students practice PCR and restriction enzyme profiling to identify a virus substrain alongside classical virology methods and tissue culture techniques. This approach introduces methods that are specific to working with animal viruses alongside fundamental molecular biology techniques that are integral to working with viruses 16.

The laboratory course in virology allows a relatively large group of students to conduct experiments using a variety of methods in virology including infection and counting methods and molecular biology techniques. This range of experimental methods gives students a broad perspective on working with viruses. Despite the variety of methods and the large number of students, students receive personal supervision that can only be achieved using the unique work station design that was developed for this lab. This approach allows for working under BSL2 conditions in parallel with BSL1 laboratories and makes maximum use of teaching resources and professional staff to enhance the learning experience of life‐science undergraduates. The course provides students with working tools that are valuable after graduation.

## Supporting information

Supporting Information
