# Supplementary material for: A biosafety level 2 virology lab for biotechnology undergraduates
Source: Biochem Mol Biol Educ. 2017 Jul 30;45(6):537–43. doi: 10.1002/bmb.21080 (PMC5697656; doi:10.1002/bmb.21080)
Supplement: Supplementary file 1 — Supporting Information [file BMB-45-537-s001.docx]

Supplementary Information

Virology Course Syllabus (Biotechnology, Hadassah Academic College)

1. Introduction:
   1. Viruses – structure and classification.
   2. Virus-host interaction
   3. Bacteriophage - structure and characteristics
   4. Virus classification according to replication mechanisms
   5. Viruses nomenclature
   6. Cell cultures: Counting and calibrating viruses. Methods according to virus' characteristics.
2. Cytopathic Viruses:
   1. RNA Viruses
      1. PicoRNA Viruses: Structure, replication and pathogenesis mechanism. Case study: Polio Virus.
      2. Influenza Virus, replication and pathogenesis.
   2. DNA Viruses
      1. Smallpox – DNA viruses that multiply in the cytoplasm?
      2. Herpes Viruses – regulation and gene expression. Replication and pathogenesis.
3. Cancer causing viruses:
   1. DNA Viruses
      1. Transformation mechanisms of DNA viruses.
      2. Herpes Virus *– in vivo* transformation ability.
      3. SV40 – the Polioma virus as a model of a transformative virus.
      4. Papilloma virus – molecular mechanism of transformation in human beings.
      5. Hepatitis B virus as an example for vaccination development in a" Virus-like Particles" approach.
   2. RNA Viruses:
      1. Retroviruses: structure, replication cycle, molecular mechanism of reverse transcription.
      2. Retroviruses as transforming agents.
      3. Immunodeficiency causing retroviruses.
4. Viral protection mechanisms:
   1. Virus-host interaction and diseases generation.
   2. Immune system defense against viruses.
   3. The interferon system.
   4. Development of anti-viral drugs.
5. Unconventional viruses – Prions.

**Virology lab – final exercise**

When plating cells for viral infection there are a few rules that must be followed. Chose the correct answer for each of the following questions. If you need to write an explanation, do not exceed the number of allocated lines.

1. I. (4 pts.) Cells must be plated in a confluence of

1. 10%-40%
2. 40%-60%
3. 70%-80%

II. (6 pts.) Explain why you chose this answer and why the other options are not suitable.

______________________________________________________________________________________________________________________________________

___________________________________________________________________

2. I. (4 pts.) For optimal experimental conditions cell must be plated:

a. A few hours before infection (on the same day).

b. A day before infection.

c. A few days before infection.

II. (6 pts.) Explain your choice.

________________________________________________________________________________________________________________________________

3. (4 pts.) During absorption, the volume of medium containing virus

a. should barely cover the cells.

b. should cover all the cells and provide nutrients for the absorption stage.

c. should cover all the cell so that no drying will occur.

(4 pts.) This is done in order

1. For the cell to be in optimal conditions, which will improve absorption chances.
2. To prevent drying of the cells.
3. To allow continues cell division.
4. To prevent viruses from having to diffuse along large distances before absorbing to the cell.

4. (6 pts.) Presence/absence of FCS during the absorption stage is important

a. For the cells to be in optimal conditions for better absorption.

b. For viral absorption to cellular receptors and not to irrelevant proteins.

c. For the cells to keep multiplying.

d. since FCS is important for blocking nonspecific proteins on the cell membrane.

5. (6 pts.) Presence/absence of polybrene during the absorption stage is important for

a. a better absorption to the cells.

b. better conditions for the cells.

c. cell continuous proliferation

d. blocking of nonspecific proteins and therefore improving absorption.

- - 1. Chose the incorrect answer:

(4 pts.) Semi solid agar should be added at the end of the absorption stage when:

a. Plaque assay is done with cytopathic viruses.

b. Focus assay is done with transduced viruses.

c. counting colored cells after infection with a defective virus with a reporter gene.

d. counting colored cells after infection with an infective virus with a reporter gene.

- - 1. (6 pts.) Explain why semi solid agar has to be added at the end of the absorption stage.
       _______________________________________________________________________________________________________________________________

- - 1. A student wants to perform an experiment. For that the student has to prepare a set of tubes with sufficient volume for a triplicate infection in each tube. Each infection should be done with 250μl of virus solution.

(10 pts.) Draw a schematic representation of five 10 fold dilutions made from an initial virus stock. As mentioned earlier, each dilution should have enough virus volume for three infections. Draw also the schematics of preparing a control dilution with no virus in the solution.

The virus stock solution is expensive. On the one hand you should not waste too much of the stock solution. On the other hand, you have to make sure there's enough solution for a triplicate infection including pipetting errors.

In your drawing label the total volume in the tube, the volume of the virus solution that was added to each tube, the volume that is transferred from one tube to the next and the leftover volume after the triplicate infection.

- - 1. (10 pts.) A student infected a plate with a defective Mulv virus that expresses GFP. For the infection the student used 500μl of a 1:10000 dilution of the virus stock solution. 24 hours after infection the student counted 40 green cells in 1mm^2^. The plae's diameter is 10cm. What is the infective viral concentration of the stock solution?
    2. A researcher wanted to check the infection efficiency of the Rous Sarcoma Virus (RSV). To this end the researcher used 25 plates. Each plate contained 250,000 cells in333μl of viral solution. The virus was diluted 10 fold for 4 times. 5 plates were prepared for each dilutions (see table). A few days later the researcher counted the number of foci in each plate. The results are summarized in the following table:

| Plate 5 | Plate 4 | Plate 3 | Plate 2 | Plate 1 | Fold dilution |
| --- | --- | --- | --- | --- | --- |
| uncountable | uncountable | uncountable | uncountable | uncountable | Viral stock |
| uncountable | uncountable | uncountable | uncountable | uncountable | 1:100 |
| uncountable | uncountable | uncountable | uncountable | uncountable | 1:1000 |
| 385 | 5 | 315 | 325 | 375 | 1:10000 |
| 37 | 29 | 33 | 35 | 31 | 1:100000 |
| 0 | 0 | 0 | 0 | 0 | Control (no virus) |

Calculate (20 pts.):

1. (6 pts.) What is the number of infective viruses in 1ml stock solution (FFU)?
2. (6 pts.) After knowing the answer for the previous question, what is the MOI of the stock solution and in the 1:1000 dilution?
3. (2 pts.) The student has to infect the cells in MOI=5. According to the information the student is interested in infecting **many/few** cells (cross out the wrong answer).
4. (6 pts.) What volume does the student have to take from the stock solution and what volume has to be taken from the absorbtion media in order to infect 250,000 cells in the desired MOI and a final volume to 333μl?

11. (10 pts. 2 for each sentence completion).

In this lab the DNA of the virus _________ is divided using______________. The DNA fragments are run on a gel of the type________________. This type of gel is used because____________________________________________. The purpose of cutting the DNA is__________________________________.
